# A top-down approach to classify enzyme functional classes and sub-classes using random forest

**DOI:** 10.1186/1687-4153-2012-1

**Published:** 2012-02-29

**Authors:** Chetan Kumar, Alok Choudhary

**Affiliations:** 1 Department of Electrical Engineering and Computer Science, Northwestern University, Evanston, IL 60201, USA

## Abstract

Advancements in sequencing technologies have witnessed an exponential rise in the number of newly found enzymes. Enzymes are proteins that catalyze bio-chemical reactions and play an important role in metabolic pathways. Commonly, function of such enzymes is determined by experiments that can be time consuming and costly. Hence, a need for a computing method is felt that can distinguish protein enzyme sequences from those of non-enzymes and reliably predict the function of the former. To address this problem, approaches that cluster enzymes based on their sequence and structural similarity have been presented. But, these approaches are known to fail for proteins that perform the same function and are dissimilar in their sequence and structure. In this article, we present a supervised machine learning model to predict the function class and sub-class of enzymes based on a set of 73 sequence-derived features. The functional classes are as defined by International Union of Biochemistry and Molecular Biology. Using an efficient data mining algorithm called random forest, we construct a top-down three layer model where the top layer classifies a query protein sequence as an enzyme or non-enzyme, the second layer predicts the main function class and bottom layer further predicts the sub-function class. The model reported overall classification accuracy of 94.87% for the first level, 87.7% for the second, and 84.25% for the bottom level. Our results compare very well with existing methods, and in many cases report better performance. Using feature selection methods, we have shown the biological relevance of a few of the top rank attributes.

## 1. Introduction

Recent advancements in sequencing technologies have seen an exponential growth in protein sequences, thus bringing to light new metabolic pathways. For many such newly found protein sequences, it is of prime interest to biologists to identify their biological function. In a biology lab, scientists conduct expensive and time consuming experiments to decipher the function of the sequences. One of the questions they often strive to address is whether the query protein is an enzyme or non-enzyme. Enzymes, as we all know catalyze biochemical reactions, but they perform this function differently using mechanisms depending on their bio-chemical properties. This has lead to the genesis of an interesting problem in Bioinformatics, i.e., given a protein sequence, how well can we classify it as an enzyme and accurately predict its function?

In light of the key biological role of enzyme proteins, the Enzyme Commission (EC) of the International Union of Biochemistry and Molecular Biology (NC-IUBMB) has created a hierarchical classification scheme based on the functional mechanism of enzymes [1]. Each enzyme is designated an EC number of the format X.Y.Z.W., where 'X' at the top of this scheme represents one of the six main classes (one-six), each further sub-divided to three levels in the hierarchy (Y.Z.W). The six main classes are Oxidoreductases (1), Transferases (2), Hydrolases (3), Lyases (4), Isomerases (5), and Ligases (6). Considering the costly experiments scientists conduct to know the enzyme mechanism, a need is felt for an automated method that can reliably predict the EC function class and thus significantly expedite experimental investigations on the query enzyme.

Enzyme function classification has engaged bioinformaticians for a considerable time now resulting in different feature extraction methods to tackle this problem. There are three prominent approaches that have been widely experimented with: first, using sequence similarity between enzymes belonging to same functional class and second protein structure comparison [2,3]. These methods have been considered inefficient since enzymes belonging to same functional class are not necessarily similar in sequence and structure [4,5]. The third approach involves representing enzymes using their sequence and structure driven features that do not use similarity.

Studies that propose methods from the third category of approaches can be found in [6-10]. Features are chosen such that they capture the bio-chemical characteristics of a protein from its protein sequence and are represented in the form of vectors. References [6,7] established that support vector machine (SVM) is useful for protein function classification showing accuracy in the range of 84-96%. This study classifies protein sequences into classes like RNA-binding, homodimer, drug absorption, drug delivery, etc., using feature vectors like amino acids composition, hydrophobicity, polarizability, and secondary structure. It thus became clear that classification using sequence features and machine learning algorithms can be useful to predict functions of proteins. Reference [9] uses 36 features drawn from enzyme protein sequences, and employs a C4.5 classifier to build the classification model. This study classified enzymes into one of the six main EC classes, achieving precision and recall in the range of 86-92%. Reference [10] uses features to represent subtle distinctions in local regions of sequence along with features as used in [9]. It applies SVM to predict the main class and reports accuracy in the range of 66.02-90.78%.

There have been efforts to predict the enzyme function to the sub-class level as well. Reference [8] uses amino acid compositions derived from sequence and employs the covariant discriminant algorithm to classify oxidoreductases (enzymes belonging to class 1) into their sub-class. Although the results are promising, this study is limited only to the scope of oxidoreductases. Reference [11] introduces a technique that uses protein sequences to compute their functional domain and PSSM matrix. It proposes a three-layer predictor model built using the optimized evidence-theoretic *k*-nearest neighbor classifier, to predict enzyme main and sub-functional class. This study does not use sequence features and achieves an overall accuracy close to 90%.

In this article, we present a new approach to predict enzyme function class and sub-class using random forest. Random forest is an ensemble-based classification and regression algorithm, considered unsurpassable in accuracy among current data mining algorithms [12]. Random forest algorithms have been applied extensively in different applications ranging from network intrusion detection [12], probability estimation [13], information retrieval, and until recently in bioinformatics [14]. Our method is based on a three-tier predicting model which when given a query protein sequence, first classifies it into an enzyme or non-enzyme, and if an enzyme it predicts the main EC function class and sub-class. To the best of authors' knowledge, this is the first article that explores the use of random forest to this particular problem. Using a unique set of sequence-driven features extracted with the aid of online tools, our model reports an overall accuracy of 94.87% for the first level, 87.7% for the second, and 84.25% for the bottom level. We also report results from a direct single-step model to predict EC sub-class, which obtained an overall accuracy of 87%. The sequence features used in our study contain the dayhoffstat value for each of 20 amino acids, which is a unique aspect of this feature set. We find that the dayhoffstat features appear in the list of top ranked attributes thus suggesting that they are important to improving classification accuracy. We also provide an analysis of one of the top ranked features, composition of Cysteine in enzyme sequences.

## 2. Materials and methods

### 2.1. Random forest

Random forest is a classification algorithm developed by Leo Breiman that uses an ensemble of classification trees [14]. Each of the classification trees is built using a bootstrap sample of the data. At every node of the tree, a candidate set of features selected from a random sub-set of the entire feature set is used to calculate the feature with the highest information gain. This strategy turns out to perform very well as compared to many other classifiers, including discriminant analysis, SVMs, and neural networks [14]. Thus, random forest uses both bagging (a successful approach for combining unstable learners) and random variable selection for tree building. Once the forest is formed, every tree classifies the instances by voting for a particular class. The class that gets maximum votes is chosen as the final classification. Random forest has several characteristics that make it well suited for enzyme function classification: (a) It runs efficiently on large datasets with many features and does not require for data to be normalized. (b) It can handle missing values. (c) Because many trees are built and each tree is effectively an independent model, the model tends not to over-fit to the training dataset.

The error rate of a random forest depends on the correlation between any two trees and the strength of each tree in the forest [12]. The random variable selection procedure applied at every split of the classification trees contributes to the low correlation between the individual trees. The strength of the tree is determined by the error rate of the tree. Reducing the correlation between the trees and increasing the strength of each tree can decrease the overall error rate of the forest. The two parameters that can help achieve this are: *mtry*, size of random sub-set of features, and *ntree*, the number of trees in the forest. Random forest error is measured in terms of out-of-bag (OOB) estimate [15]. Increasing ntree reduces the OOB error rate of the forest as it decreases the correlation between individual trees and the possibilities of over-fitting. *mtry *should be a value much smaller than the total number of features. In most cases, an optimum value between *ntree *and *mtry *results in the lowest OOB error and higher accuracy.

To improve the classification accuracy, we have optimized the parameter values at every level of the model. In this article, we also present results obtained using a direct single-step model, in which a model built using random forest is trained on enzymes labeled with their sub-classes and tested on an independent set. The architecture of the two models is explained in the next section.

### 2.2. Model description

**Figure 1:**
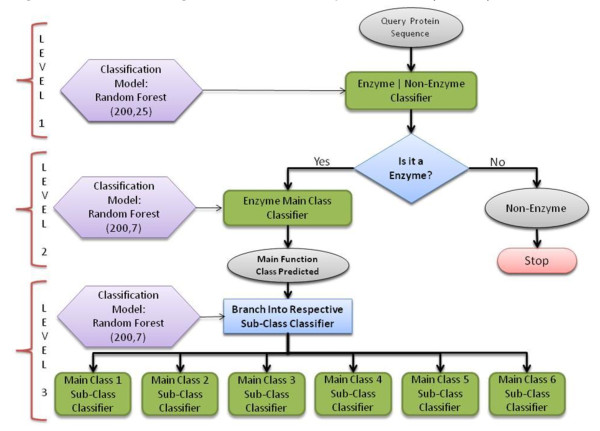
**A flowchart diagram of the three-tier top down model (Model 1)**.

In this article, we focus on the three-tier top-down model to predict enzyme function till the sub-class level and also share results from a direct one-step approach to predict the same. The former model comprises of three layers: the first layer classifies enzymes and non-enzymes, the second predicts the main function class of the classified enzymes and the third layer predicts their sub-class. Each of the three layers is built using a random forest classifier with parameter values optimized to achieve highest accuracy possible. Figure 1 illustrates the design of the model with optimized parameter values at each level.

A diagram showing different components of the three-tier model. The first level classifies enzymes from non-enzymes. This model has been trained using a random forest with parameter values *mtry *= 25 and *ntree *= 200. Level 2 classifies enzymes into their main function class, while level three classifies the enzymes whose main class is predicted in level 2, into the sub-classes. There are six classifiers in level 3, each for the corresponding main class. The level three classifier is built using a random forest with parameter values identical to level 2, i.e., *mtry *= 7 and *ntree *= 200.

**Figure 2:**
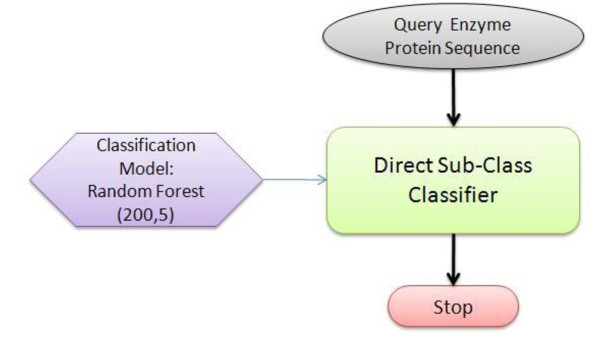
**A flowchart diagram of direct sub-class level prediction model (Model 2)**.

The second of the two models is a direct one-step approach to predict the sub-class function level (see Figure 2). This model was built by training random forest using instances of enzymes labeled with their sub-class. Once, the parameter values were optimized, the model was tested on an independent test set. Later sections discuss the comparison of results from the two approaches discussed above.

This model uses a query enzyme sequence and directly classifies into the sub-class. This model has been built using a random forest classifier with optimized parameter values, *mtry *= 7 and *ntree *= 200. These values correspond to the minimum OOB error rate obtained using this classifier.

### 2.3. Sequence extraction

We extracted protein sequences of enzymes from the enzyme repository of SWISS-PROT database [16]. Research in machine learning has proved that imbalance in class size can be an obstacle in building an accurately predicting model [17]. Hence, the number of sequences extracted from every main class was kept well balanced. Since, each main class has many sub-classes, we randomly extracted sequences such that they are well distributed over the latter. The next step was to remove identical sequences present in each main class. For this, we used CD-HIT, a program that removes redundant sequences, given a sequence identity threshold, which we set to 100% [18]. Table 1 summarizes the distribution of sequences across all the main classes and sub-classes. We selected sequences from only those sub-classes that contained significant number of sequences (> 200 sequences). The third column represents the sequences after removing all identical sequences.

**Table 1:** Distribution of sequences across different classes in training and test data combined together

Class	Sub-classes	Number of sequences
1 Oxidoreductases	1.1, 1.2, 1.3, 1.4, 1.5, 1.10, 1.16	986
2 Transferases	2.1, 2.2, 2.3, 2.4, 2.5, 2.6, 2.7, 2.8	734
3 Hydrolases	3.1, 3.2, 3.3, 3.4, 3.5, 3.6, 3.7, 3.8, 3.11	674
4 Lyases	4.1, 4.2, 4.3, 4.4, 4.6, 4.99	828
5 Isomerases	5.1, 5.2, 5.3, 5.4, 5.5	664
6 Ligases	6.1, 6.2, 6.3, 6.4	845

The sequences extracted from SWISS-PROT enzyme database are spread over a total of 40 sub-classes. Sequences have been extracted from the sub-classes having the largest bank of sequences. The number of sequences shown represent sequences with 100% reduced identity.

### 2.4. Feature representation

**Figure 3:**
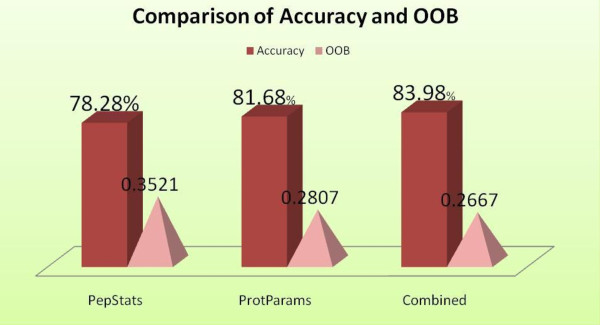
**Accuracy and OOB error obtained using features from PepStats, ProtParams and combined features from the two tools**.

**Figure 4:**
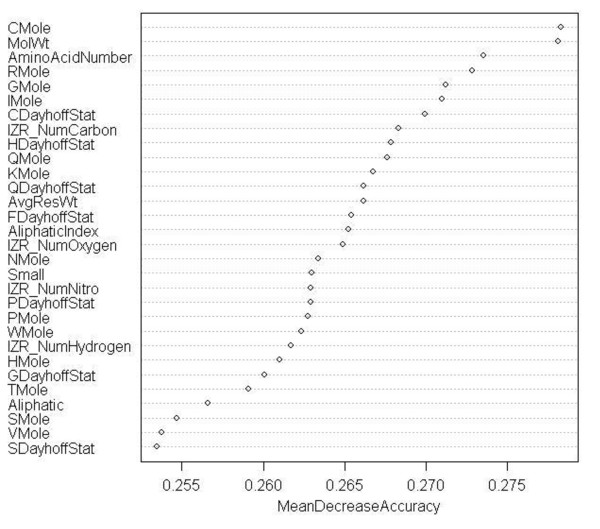
**Mean decrease accuracy of top attributes for predicting main class of enzymes, computed using variable importance**. Model 2 random forest classifier OOB error for different *ntree *and *mtry *values.

To extract sequence-derived features, we used two online tools, EMBOSS-PEPSTAT: an online tool that generates a list of 61 feature values for a given sequence [19], and ProtParams: an online tool that computes values for 36 sequence features [20]. PEPSTATS generates values for features such as molecular weight, iso-electric point, amino acid composition, aliphatic amino acids, molar compositions of aromatic, polar, non-polar, charged, basic, and acidic amino acids. A unique aspect of this tool is that it provides the dayhoffstat value for every amino acid present in the sequence. As defined by EMBOSS, dayhoffstat is the amino acid's molar percentage divided by the dayhoff statistic. The dayhoff statistic is the amino acid's relative occurrence per 1000 amino acids normalized to 100 [19]. ProtParams, on the other hand, does not compute dayhoffstat values. However, it provides for feature values such as number of negatively or positively charged residues, number of carbon, hydrogen, nitrogen, oxygen and sulfur atoms, GRAVY, theoretical-pI, and aliphatic index. The use of these features is well reasoned and motivated in previous studies [21,22]. From our experiments, we find that a union of the features of ProtParams and PEPSTATS delivers better accuracy in comparison to using only one of the two feature sets. Figure 3 presents a comparison of the OOB error and accuracy for three cases obtained using a random forest classifier with default settings (*mtry *= 7, *ntree *= 10). Unique features from both tools such as dayhoffstat and number of carbon atoms play a significant role in enhancing the classification accuracy. This is corroborated by the fact that they appear in our analysis of the top predicting attributes (Figure 4).

The classifier used is random forest with parameter values, i.e., *ntree *= 10, *mtry *= 7. PepStats, an online sequence analysis tool, computes values for 61 sequence features, while ProtParams computes for 36 features. The classification result obtained after taking a union of the features from the two tools is shown in the third bar. Some of the features that are unique to each tool help in improving the accuracy and reducing OOB error.

### 2.5. Dataset preparation and tools used

We selected a total of 2400 non-enzyme sequences and 4731 enzyme sequences. For level 1 experiment, we randomly selected 2400 enzyme sequences against an identical number of non-enzyme sequences. For levels 2 and 3 experiments, we divided the 4731 enzymes equally into training and test data, each containing 2366 and 2365 instances, respectively. The distribution of sequences across different classes was kept equivalent in both test and train data, as can be seen from Table 1. We did not normalize the feature values. WEKA, a widely used open source tool in machine learning was used to carry out all experiments [23]. We used Rattle, to perform feature selection using variable importance method [24].

## 3. Results

### 3.1. Results from experiments using model 1

**Figure 5:**
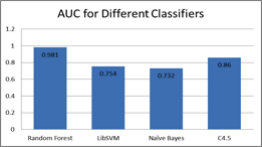
**Area under the ROC curve for different classifiers to predict enzyme main class**.

First, experiments were carried out with different classifiers to identify the best classifier for our dataset. We carried out tenfold cross-validation experiments between LibSVM [25], NaiveBayes, C4.5 [26], and Random Forest, with default settings and parameters for all, as set by Weka. The experiment was performed at level-2, i.e., to predict the main class of the enzymes. Figure 5 illustrates the area under the ROC curve for the four different classifiers. Random forest out-performed all the remaining classifiers by recording the highest area under the curve.

Figure 5 plots the area unde the ROC curve reported after running Weka on the different classifiers. The experiment was performed on enzyme sequences to predict their main class. Random forest recorded the highest area as compared to LibSVM, Naïve Bayes and C4.5.

### 3.2. Level 1: enzyme | non-enzyme classification

**Figure 6:**
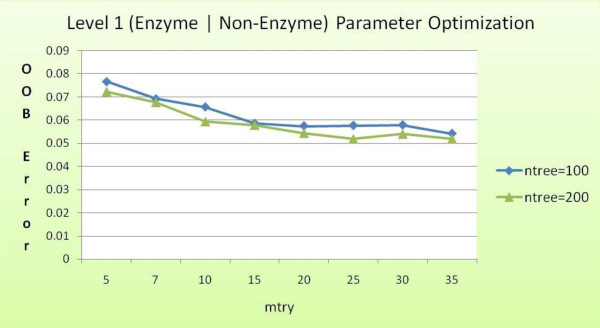
**Level 1 OOB error for different values of *mtry *and *ntree***.

Level 1 of the model classifies enzyme protein sequences from non-enzyme protein sequences. We performed tenfold cross-validation experiments on a dataset containing values for all 73 features extracted from 2400 enzyme and non-enzyme protein sequences, a total of 4800 sequences. We first sought to optimize the two random forest parameters, *ntree *and *mtry*. Figure 6 provides OOB error estimates for varying values of *ntree *and *mtry*.

This graph shows OOB error obtained for different runs of the random forest classifier during its training phase. As the values of *mtry *and *ntree *are changed, the OOB error also varies. With increasing *mtry *and *ntree*, the error appears to decline till a certain value of *mtry*, i.e., 25 is reached. Hence, *mtry *= 25 and *ntree *= 200 are selected as values for the parameters for the level 1 classifier.

The least OOB error is obtained when *ntree = 200 *and *mtry = 25*. We anchor these parameter values for level 1 classifier. Table 2 summarizes the results obtained from a tenfold cross-validation experiment. The overall accuracy obtained is 94.87%, with an OOB error of 0.056. This result compares quite favorably with other articles [11,27] that report an overall accuracy of approximately 75% (using neural network) and 91.3%, respectively.

**Table 2:** Tenfold cross-validation results obtained from experiment to classify enzyme and non-enzyme protein sequences

Protein type	Sequences	Correctly predicted	Precision	Recall	Accuracy
Enzyme	2399	2287	94.50%	95.30%	95.33%
Non - Enzyme	2399	2265	95.30%	94.40%	94.41%
Overall	4798	4552	-	-	94.87%

### 3.3. Level 2: enzyme main function class classification

**Figure 7:**
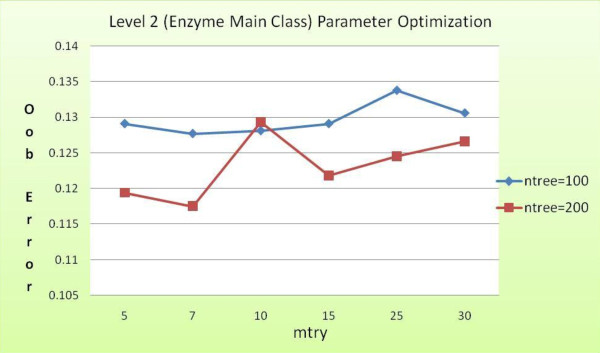
**Level 2 OOB error for different values of *mtry *and *ntree***.

Using a training and test data consisting of 2366 and 2365 instances, respectively, the second layer in the model classifies the test set of enzyme sequences into one of the six EC main function classes. We carried out several runs of the random forest classifier to obtain the optimal values of *ntree *and *mtry*, the results of which are shown as a graph in Figure 7. As can be seen in the figure, the lowest OOB error (approx. 0.117) is obtained when *ntree *= 200 and *mtry *= 7, respectively. Table 3 summarizes the classification results from level 2 classifier built using these parameter values.

**Table 3:** Classification results on test data for main enzyme class classification using level 2 classifier

Class	Total enzymes	True positive	False positive	Precision (%)	Recall (%)	ROC area
1	493	436	57	88.40	88.40	0.94
2	367	302	49	86	82.30	0.92
3	337	297	66	81.80	88.10	0.95
4	414	371	53	87.50	89.60	0.95
5	332	281	32	89.80	84.60	0.94
6	422	387	34	91.90	91.70	0.96
Overall	2365	2074	291	87.70	87.70	0.94

Figure 7 shows OOB error obtained from different runs during training phase of the level 2 random forest classifier. The least value of OOB error is obtained when *mtry *= 7 and *ntree *= 200. Hence, these values are selected for the parameters for the level 2 classifier.

The overall classification accuracy achieved was 87.7%, with 2074 enzymes being correctly classified into their main function class out of a total of 2365 instances. This accuracy has been attained by a combination of 73 sequence driven features (union of PepStats and ProtParams features) and random forest classifier with optimal parameter values. In comparison to [9] that applies features from ProtParams and [10] which uses those from PepStats, respectively, this is a significant improvement in accuracy. Further, the dataset used in this study comprises of a total of 4731 enzyme sequences spread over 39 sub-classes. The dataset used in [9] contains 780 enzymes spread over 18 sub-classes. Random forest achieves a higher accuracy despite a wider distribution of enzyme proteins. These results substantiate the application of random forest to classification problems in bio-informatics.

Using random forest, we also carried out tenfold cross-validation experiments on all of the 4731 sequences with *mtry *= 7 and *ntree *= 200. We found 4171 or 88.16% of the sequences to be correctly classified into their main enzyme class, with an overall root mean squared error of 0.1992. Table 4 summarizes the results from this experiment.

**Table 4:** Results of tenfold cross-validation experiment performed using Model 1 to predict main enzyme class

Class	Total sequences	True positive	False positive	Precision rate (%)	Recall (%)
1	986	878	115	88.40	88.70
2	734	602	95	86.40	84.10
3	674	610	124	83.10	86.60
4	828	737	81	90.10	89.60
5	664	558	75	88.20	86
6	845	786	70	91.80	92.40
Overall	4731	4171	560	88.20	88.20

The next step is to predict the sub-class for the enzymes. To do this, first we collected the enzymes classified into their respective main classes, into different files. For example, we took all enzymes classified as belonging to class 1, and used them as a test dataset for the level three classifier. We repeated this process for all six classes. The six level three classifiers were trained using corresponding main class instances from level two training data. As an illustration, the level three, sub-class 1 classifier was trained using main class 1 instances that were used for level two training, but are now labeled with their corresponding sub-class.

### 3.4. Level 3: enzyme sub-class function classification

In level three of the model, we classify enzymes whose main class has been predicted, into the sub-class that they might belong to. There are six random forest classifiers in this stage, each to predict the sub-class for enzymes under the corresponding main class. We used the same parameter values as used in level two for all six classifiers of level three, i.e., *ntree *= 200 and *mtry *= 7. This is because we did not see a big difference in OOB error even after varying values of *mtry *between 5 and 25.

The level two classifier also generates false positives, as shown in Table 3. If we consider class 1 only, false positives here are the enzymes that are classified as class 1 but actually belong to other classes like 2 or 3. As a result, as an example enzymes might get wrongly assigned a sub-class label of 1.2, which in reality is 2.2. Hence, we need to account for false positives as errors while reporting the classification accuracy of level three. Table 4 carries a column titled carry over false positives. These are the enzymes wrongly predicted as belonging to the respective main class. For class 1, there are 57 such enzymes that we need to account and distribute across the sub-classes of class 1. We factor the number of carry over false positives by the number of test sequences in each sub-class. For instance, sub-class 1.1 has 81 enzymes while 1.16 has 35 enzymes, hence number of carry over false positives to 1.1 is twice that for 1.16, i.e., 10 and 5, respectively. We calculate new values for precision by the addition *f *carry over false positives and false positives reported in the experiment. The formula we use is as follows:

$$\text{Precision}=\frac{\text{TruePositves}}{\text{TruePositves}+\text{FalsePositives}+\text{CarryOverFalsePositives}}$$

False negatives are instances of class 1, for example, that get wrongly classified as being in class 3, and hence the sub-class will also be wrongly identified, say 3.1 instead of 1.2. Just like precision, we calculate new values for recall that take into account the false negatives generated in level two. New recall values are calculated using the formula given below:

$$\text{Recall}=\frac{\text{TruePositves}}{\text{TruePositves}+\text{FalseNegatives}+\text{CarryOverFalseNegatives}}$$

**Figure 8:**
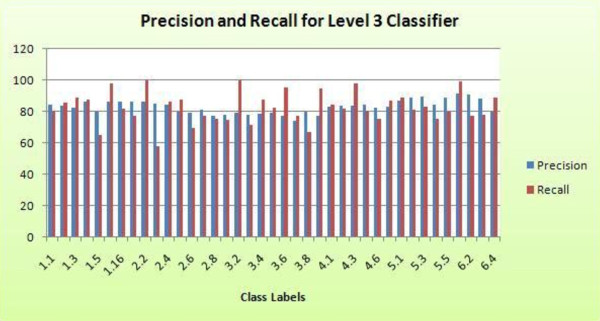
**Graph showing precision and recall for all sub-classes obtained using level 3 random forest classifier**.

Table 5 and Figure 8 provide a quantitative estimate of the performance of random forest in predicting the sub-class of the enzymes. The overall precision and recall when we do not account for carry over false positives and false negatives is 95.67 and 95.34%, respectively, and after incorporating these errors, the overall precision and recall falls to 83.01 and 82.67%, respectively. Precision and recall across all sub-classes ranges from 74.07 to 91.19% and 57.5 to 100%, respectively. From the results, we can deduce that at level 2, if the classifier correctly predicts the main class, there is 95% probability that level three will correctly identify the sub-class. However, if it does not predict the main class correctly, this probability drops to 83%. This deduction is also established by the correlation between the ROC area for the main class and the corresponding sub-classes. From Table 3 we can see that the ROC Area is highest for class 6 and lowest for class 2. When we look at the ROC area for their corresponding sub-classes, in Table 5 we notice that the sub-classes of class 6 have higher ROC area as compared to sub-classes of class 6. Summarizing the results, it is clear that the three layer model has achieved highly promising results, with the capability to correctly predict till the sub-class level with 83% accuracy.

**Table 5:** Level 3 classification results for sub-classes of all six main classes

Class	Size	False positives	Carry over false positives	Precision	New precision	Recall	Carry over false negatives	New recall	ROC area
1.1	81	4	10	95.06	84.61	95.1	15	80.20	0.96
1.2	81	5	10	93.98	83.87	96.3	10	85.71	0.96
1.3	81	7	10	91.76	82.11	96.3	7	88.63	0.99
1.4	81	2	10	97.44	86.36	93.8	6	87.35	0.96
1.5	34	3	5	91.18	79.48	91.2	14	64.58	0.89
1.10	43	0	7	100	86	100	1	97.72	0.98
1.16	35	0	5	100	86.48	91.4	4	82.05	0.98
2.1	38	0	6	100	86	97.37	10	77.08	0.96
2.2	45	0	7	100	86.5	100	0	100.00	0.97
2.3	23	0	4	100	85.2	100	17	57.50	0.96
2.4	44	1	7	97.73	84.3	97.72	6	86.00	0.99
2.5	43	4	7	91.49	79.6	100	6	87.75	0.98
2.6	36	3	6	91.89	79.1	94.44	13	69.38	0.95
2.7	36	1	6	96.77	81.1	83.33	3	76.92	0.94
2.8	37	4	6	89.47	77.3	91.89	8	75.55	0.89
3.1	35	1	8	96.97	78.05	91.43	8	74.41	0.97
3.2	45	2	10	95.74	78.95	100		100.00	0.95
3.3	34	1	8	96.97	78.05	94.18	11	71.11	0.94
3.4	46	2	10	95.56	78.18	93.48	3	87.75	0.93
3.5	42	0	10	100	79.17	90.48	4	82.60	0.97
3.6	44	3	10	93.62	77.2	100	2	95.65	0.97
3.7	20	3	4	86.96	74.07	100	6	76.92	0.92
3.8	13	1	2	92.31	80	92.3	5	66.66	0.90
3.11	18	1	4	94.44	77.27	94.45		94.44	0.97
4.1	76	4	11	94.87	83.15	97.37	12	84.09	0.99
4.2	78	4	11	95	83.51	97.44	15	81.72	0.98
4.3	90	4	13	95.7	83.97	98.89	1	97.80	0.97
4.4	41	1	6	97.37	84.09	90.24	5	80.43	0.97
4.6	42	2	6	94.87	82.22	88.1	7	75.51	0.96
4.99	43	2	6	95.24	83.33	93.02	3	86.95	0.97
5.1	83	2	10	97.59	87.1	97.6	8	89.01	0.98
5.2	39	1	4	97.5	88.63	100	9	81.25	0.99
5.3	78	1	8	98.73	89.65	100	16	82.97	1.00
5.4	40	2	5	94.87	84.1	92.5	9	75.51	0.97
5.5	40	0	5	100	88.63	97.5	9	79.59	0.98
6.1	207	2	18	99.04	91.19	100	1	99.51	1.00
6.2	73	1	6	98.61	91.02	97.26	19	77.17	0.98
6.3	72	3	6	95.65	88	91.67	13	77.64	0.95
6.4	35	5	3	86.49	80	91.43	1	88.88	0.94
Overall	2072	82	290	95.67	83.01	95.34	287	82.670	0.96

Carry over false positives and negatives from level two classifier experiments are taken into account while calculating precision and recall in level three. The distribution of the carry over false positives is factored by the number of test sequences in the respective sub-classes, in order to conserve the sequence distribution

Precision and recall for most sub-classes is around similar range, besides seven sub-classes that have a higher recall. Minimum precision is 74.07% (sub-class 3.7) while minimum recall is 57.05% (sub-class 2.3).

### 3.5. Results from experiments using model 2

**Figure 9:**
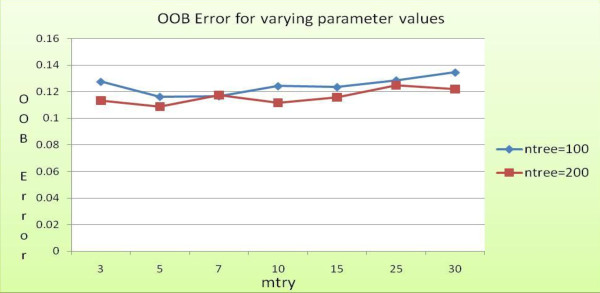
**Model 2 random forest classifier OOB error for different *ntree *and *mtry *values**.

Model 2 (see Figure 2) is a direct single step approach to predicting the sub-class of enzymes. As in previous cases, we first sought to find optimal values of the random forest parameters. We carried out a tenfold cross-validation experiment. The random forest classifier reports the lowest OOB error when *mtry *= 5 and *ntree *= 200 (see Figure 9). Using these values, the results from the experiment are summarised in Table 6.

**Table 6:** Performance of random forest classifier using Model 2, direct sub-class classification approach

Class label	Instances	True positive	Precision (%)	Recall (%)
1.1	192	157	83.51	81.77
1.10	88	87	93.55	98.86
1.16	74	65	89.04	87.84
1.2	179	169	88.02	94.41
1.3	176	162	94.74	92.05
1.4	175	162	94.74	92.57
1.5	97	78	81.25	80.41
2.1	96	80	95.24	83.33
2.2	91	90	70.87	98.9
2.3	80	39	60.94	48.75
2.4	98	83	91.21	84.69
2.5	97	86	88.66	88.66
2.6	96	82	82.83	85.42
2.7	110	68	93.15	61.82
2.8	93	74	94.87	79.57
3.1	87	71	77.17	81.61
3.11	36	33	80.49	91.67
3.2	89	85	86.73	95.51
3.3	87	76	87.36	87.36
3.4	95	86	94.51	90.53
3.5	91	85	91.4	93.41
3.6	92	88	92.63	95.65
3.7	55	43	91.49	78.18
3.8	36	31	79.49	86.11
4.1	172	159	90.34	92.44
4.2	185	147	89.63	79.46
4.3	183	180	91.84	98.36
4.4	91	77	91.67	84.62
4.6	97	85	91.4	87.63
4.99	93	87	92.55	93.55
5.1	179	168	85.28	93.85
5.2	96	86	83.5	89.58
5.3	188	154	84.15	81.91
5.4	98	77	92.77	78.57
5.5	99	84	85.71	84.85
6.1	418	416	93.91	99.52
6.2	183	156	81.68	85.25
6.3	170	151	87.79	88.82
6.4	82	70	80.46	85.37
Overall	4748	4190	87.35	86.74

Figure 9 shows OOB error obtained from different runs of the random forest classifier during training phase. The least value of OOB error is obtained when *mtry *= 5 and *ntree *= 200.

The overall precision and recall obtained using Model 2 is 87.35 and 86.74%. Precision ranges from 60.94 to 95.24% while recall lies in the ranges 48.75-99.52%. We also tested model 2 by introducing 784 non-enzyme sequences into the dataset. For this, we conducted another tenfold cross-validation experiment using the same values for *mtry *and *ntree*, 5 and 200, respectively. Random forest correctly classified 86% of the sequences, where the precision and recall of the non-enzyme class was 87.4 and 86.6%, respectively. This is lower than the results from level-1 of model-1 which reported around 94% accuracy.

## 4. Discussion

Although both models 1 and 2 report promising results, comparing favorably well with other published studies [8-11], the precision and recall obtained using model 2 (87.35 and 86.74%, respectively) is higher in comparison to model 1. If we only look at the minimum precision and recall values, model 2 reports 60.94 and 48.75%, respectively, both for sub-class 2.3. Through model 1, the precision for the same class 2.3 is 85.2% while recall is 57.5%. Model 1 has the advantage that if we only consider the instances whose main class is predicted correctly, the precision for predicting the sub-class is very high, almost 95%. This result leads us to reliably conclude that the set of features extracted from enzyme protein sequences capture rich information about the functional mechanism of the enzyme, down to the sub-class level. Further, model 1 is designed with the objective of segregating enzymes from non-enzymes, and subsequently predicting main and sub-class of the enzymes. It can hence be applied to any generic sequence. This could be helpful to biologists for they would first want to know whether a query protein sequence is an enzyme or not. Model 2 on the other hand proves to be more effective to sequences that are already known to be enzymes.

## 5. Ranking attributes

In a classification problem, ranking the features is often of interest as it tells us which features are strong predictors. Reference [17] has indicated that it is possible not all features from a protein sequence are strong predictors and hence many might contribute to noise. In random forest, importance of features is computed using a method called variable importance [15]. This method provides two indices to quantify which features are most informative, i.e., exhibit strong characteristics associated with enzyme function classes: mean decrease in accuracy and the gini index. Mean decrease in accuracy is considered more reliable and accurate than the gini index [12]. Hence, we used the former to report the strong predictors. Since WEKA does not provide the variable importance feature for random forest as yet, we used Rattle for this purpose, a data mining tool developed by Dr Graham Williams [24]. The experiment we performed was to compute the top predicting attributes for the enzyme main class by way of tenfold cross validation. Figure 4 lists the top features computed by the variable importance method.

Figure 4 shows the top predicting attributes in decreasing order of accuracy. CMole represents the Cysteine percentage composition in the protein sequences. MoltWt is the molecular weight. HDayhoffStat is dayhoffstat value for Histidine in the enzyme protein sequences.

**Figure 10:**
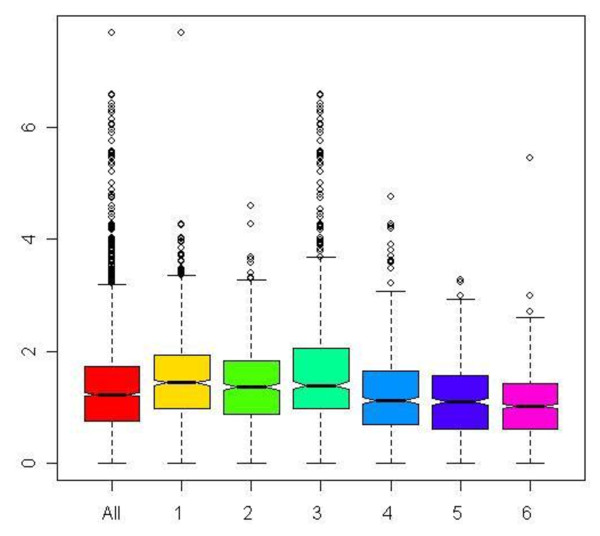
**Box plot diagram showing distribution of cysteines across the six main enzyme function classes**.

From the figure we can see that Cysteine amino acid (CMole) has the highest prediction accuracy, followed by molecular weight and amino acid number. A box plot diagram in Figure 10 provides the relative distribution of cysteines in the six main function classes. Sequences that belong to class 3, i.e., hydrolases have the highest median composition of cysteines and highest upper quartile value. We verified this information with published studies and found that studies carried out in [28] report high conservation of cysteines in glycosyl hydrolase family, which are enzymes from class 3. Reference [29] has analyzed proteins from this family and also reports high cysteine conservation in glycoside hydrolases. Hence, results from this experiment might indicate that composition of cysteines is higher in hydrolases. This would however need to be validated and verified with biological experiments.

Figure 10 showing class 3, i.e., Hydrolases to have the highest median and upper quartile percentage composition of Cysteines.

We also noted the top predicting attributes for enzyme versus non-enzyme classification and sub-class level classification. First, for the enzyme versus non-enzyme classification: CMole was not quite the top predicting attribute, although its Mean Decrease Accuracy figure was about the same (0.26). Molecular weight (MolWt.) was the top predicting attribute for this experiment, with a mean decrease accuracy of 0.36. Next, for the enzyme sub-class level classification: CMole, AminoAcidNumber and MolWt were amongst the top four predictors, with the mean decrease accuracy ranging between 0.32 and 0.33.

## 6. Conclusions

Enzyme function classification is a challenging problem, and sequence features alone will not be enough to accurately predict enzymatic mechanisms. However, using a unique set of features extracted from sequence and an efficient classifier, random forest, we have demonstrated that sequence features do capture rich bio-chemical information about an enzyme and if coupled with structural characteristics, can contribute to a more robust and accurately predicting model. By using 73 different features extracted using EMBOSS PEPSTAT and ProtParams tool, we have tried to highlight how existing tools can be re-used and extended to address interesting problems in Bioinformatics. The results from the experiments demonstrate the useful application of random forest for multi-class problems like enzyme function classification. The random forest classifier achieved a high accuracy on a widely distributed and reasonably large dataset. Further, our analysis of top rank features suggests that percentage composition of cysteines can be important in enzyme function classification. The datasets are available online for other groups to experiment and could prove to be useful for extracting interesting information about enzymes, especially with regard to the features that we have used.

## Competing interests

The authors declare that they have no competing interests.
